# Incorporating Colour Information for Computer-Aided Diagnosis of Melanoma from Dermoscopy Images: A Retrospective Survey and Critical Analysis

**DOI:** 10.1155/2016/4868305

**Published:** 2016-12-19

**Authors:** Ali Madooei, Mark S. Drew

**Affiliations:** School of Computing Science, Simon Fraser University, Burnaby, BC, Canada

## Abstract

Cutaneous melanoma is the most life-threatening form of skin cancer. Although advanced melanoma is often considered as incurable, if detected and excised early, the prognosis is promising. Today, clinicians use computer vision in an increasing number of applications to aid early detection of melanoma through dermatological image analysis (dermoscopy images, in particular). Colour assessment is essential for the clinical diagnosis of skin cancers. Due to this diagnostic importance, many studies have either focused on or employed colour features as a constituent part of their skin lesion analysis systems. These studies range from using low-level colour features, such as simple statistical measures of colours occurring in the lesion, to availing themselves of high-level semantic features such as the presence of blue-white veil, globules, or colour variegation in the lesion. This paper provides a retrospective survey and critical analysis of contributions in this research direction.

## 1. Introduction

Image-based computer-aided diagnosis systems have significant potential for screening and early detection of melanoma. At present, digital dermoscopic images seem to be the most promising source for automatic or computer-aided melanoma diagnosis [1]. Automatic dermoscopy image analysis is mainly concerned with two tasks: (i) identifying dermoscopic features in each image and (ii) associating those features with a diagnosis (image classification of skin lesions). Both tasks are examples of visual recognition that itself is a classical problem of computer vision. The organization of a computer vision system is highly application-dependent. For automatic dermoscopy image analysis, a typical system pipeline involves preprocessing, lesion segmentation, feature extraction, and classification. These steps are briefly described next. For a detailed survey, the interested reader is referred to [2, 3].

(i) *Preprocessing* used for dermoscopy images is of many kinds, including but not limited to image enhancement (such as colour correction, shading removal, and contrast adjustment), colour space transformation, and removal of artifacts (such as hairs, ruler markings, air bubbles, black frames, and ink markings). Among these, previous efforts have mostly been focused on the development of hair removal algorithms.
(ii) *Lesion segmentation* involves isolating skin lesions from normal skin surrounding it. It is important for two reasons: Firstly, the lesion boundary provides important information for accurate diagnosis. For example, border asymmetry or irregularity can signal malignancy. Secondly, it is a common practice to perform feature extraction for the lesion area only, masking the healthy skin surrounding it. Therefore, representativeness of image features depends on the accuracy of the segmentation. Lesion segmentation is one of the most studied topics. It poses a challenge for several reasons, including low contrast between the lesion and its background and the presence of artifacts.
(iii) *Feature extraction* involves detection, description, and representation of image features. The concept of image feature is very general but, in the context of dermoscopy image analysis, it can be defined as computing abstractions of information contained within the image that is relevant for diagnosis. This abstraction can be made at different levels (local versus global, low-level versus high-level) depending on how subsequent algorithms operate. For example, primitive or low-level features such as the distribution of image intensity, texture, or colour may be used for solving the computational task related to the diagnosis of a medical condition. A common trend in recent years is in developing high-level semantic (clinically meaningful) visual features that can be used by both clinicians and computer programs for computer-assisted diagnosis.
(iv) *Classification* is usually the final step. The output of lesion classification depends on the application but it is often an estimate of the probability of malignancy. To this aim, a typical approach would be based on the classical paradigm of supervised machine learning that requires training images. Classification might be preceded by* feature selection* which is the process of selecting a subset of relevant features for use in model construction. In many cases, the derivation of high-level semantic features also requires the use of classification (or other machine learning techniques).

Colour features are among the most widely used features in image analysis tasks. Colour has been arguably the most widely used feature in dermoscopy image analysis as well. This is not a surprise since colour is an essential part of most clinical methods for screening of skin lesions. The presence of multiple colours with an irregular distribution highly suggests malignancy. Among dermoscopic features of melanoma, colour has a substantial role too. The presence of certain shades of blue in a lesion implies the possible diagnosis of melanoma more so than a lesion manifesting only different shades of brown. Thus, the correct clinical diagnosis of a pigmented skin lesion requires accurate evaluation of its colouration in terms of its shades and distributions. This paper is aimed at reviewing the literature on incorporating colour features for computer-aided diagnosis through dermoscopy image analysis.

The studies cited in this review were retrieved through a careful literature search using several databases, namely, IEEE* Xplore* (http://ieeexplore.ieee.org/), Springer* Link* (http://link.springer.com/), Science Direct (http://www.sciencedirect.com/), Wiley Online Library (http://onlinelibrary.wiley.com/), Web of Science (http://webofscience.com/), PubMed (http://www.ncbi.nlm.nih.gov/pubmed), and Google Scholar (https://scholar.google.com/). One of the challenges of finding relevant literature, as it is with cross-disciplinary research, is to survey a wide array of topic areas from clinical practice to scientific research that includes specialized journals to multidisciplinary conferences. The initial search strategy included keyword search in combination with category filtering and retrieved more than 1500 articles. These articles were archived using* Zotero*, a web browser plug-in for gathering and organizing research resources. Using Zotero, duplicated results were removed. Moreover, through analysis of articles' titles and keywords, all results pertaining to the following categories were manually removed:

(i) Studies that used imaging modalities other than dermoscopy or clinical photography, including confocal microscopy, histopathology, laser Doppler, MRI, multiphoton laser tomography, ultrasound, near infrared, (any type of) spectroscopy, fluorescence imaging, stereo imaging, and multi- or hyperspectral imaging.
(ii) Studies that did not focus on computer-aided diagnosis through image analysis. For example, clinical trials, case studies, or studies on genome, biomarkers, bioprofile, and proteins were all removed.
(iii) Studies which were not aimed at diagnosis of skin cancer. For example, topics of “wound healing evaluation,” “assessment of burn scars,” “erythema measurement,” and “detection of psoriasis” were removed.

The initial retrieval set was reduced to about 600 articles at this stage. This reduced set was carefully reviewed to further identify those studies using colour features for computer-aided diagnosis through dermoscopy image analysis. At the end, more than 100 articles (published from 1989 to 2015 in more than 20 different publications) were selected. This final set is critically reviewed in the following sections.

## 2. Clinical Background

Dermoscopy (a.k.a. epiluminescence microscopy, dermatoscopy, or amplified surface microscopy) uses a handheld microscope called a dermatoscope (or dermoscope) for in vivo examination of skin lesions. It can be used to differentiate most lesions of the skin from melanoma [4]. In the hands of experienced users, dermoscopy can improve the clinicians' diagnostic accuracy by 10% to 30% (depending on the type of skin lesion) [5, 6] and increases the physicians' confidence in their clinical diagnoses [7, 8], thus significantly reducing unneeded biopsies [9].

The dermoscopic diagnosis of pigmented skin lesions is based on various analytic approaches or algorithms that have been set forth in the last few decades. The common denominators of all these diagnostic methods are particular dermoscopic features (such as colours and patterns) that represent the backbone for the morphologic analysis of pigmented skin lesions.

### 2.1. Dermoscopic Colours

Skin colour is the result of the interaction of light with skin pigments (melanin, hemoglobin, and keratin) and other materials (collagen, serum in crusts, etc.). Dermatoscope reveals a wider range of colours than those that can be seen with the naked eye. Common colours under dermatoscope include various shades of brown, blue, black, white, and red. There is more of these colours than those that meet the eye; colours can convey critical morphologic information. For instance, shades of blue might be caused by the presence of melanin pigment in the deeper layers of skin (deeper than expected) which itself can be a clue to invasive melanoma. Among other colours, shades of red can be associated with increased vascularization, dilatation of blood vessels, and bleeding within the lesion.

Melanin is the main chromophore in pigmented skin lesions. Melanin is black but, depending on how deeply and densely in the skin it is located, it might be perceived differently. If melanin is situated in the stratum corneum or immediately beneath it, the lesion would appear black on dermoscopy. Melanin at the dermoepidermal junction would be perceived as light brown to dark brown, depending on its density. Melanin in the dermis results in shades of grey to blue (when located in the deeper dermis).

The other important determinant of lesion colouration is hemoglobin in red blood cells which results in shades of pink and red (depending on the vascular volume). Poorly oxygenated blood might be perceived as purple, and congealed blood (thrombus) often appears as jet black. The stratum corneum, devoid of blood and melanin, appears yellow. Orange hues are due to serum present in erosions or superficial ulcerations. White colour is due to depigmentation, fibrosis, alterations in the collagen matrix, or keratin within cysts.

Benign lesions (usually) reveal only one or two colours whereas melanomas (frequently) reveal three or more colours. Thus, the number of colours present in a lesion can also help differentiate many benign nevi from melanomas. It is important, however, to appreciate that not all colours have the same impact on the diagnosis of melanoma. The presence of red, white, and/or blue-grey colours in a lesion implies the possible diagnosis of melanoma more so than a lesion manifesting only different shades of brown. Moreover, distribution of colours in melanoma are often focal, asymmetrical, and irregular. Thus, the correct clinical diagnosis of a pigmented skin lesion requires an accurate evaluation of its colouration (concerning the shades and the distribution of colours).

In addition to common colour names, dermatologists often use other descriptive terms to describe skin colouration. These include

(i) hyperpigmentation: skin colour that is darker than normal. This is usually associated with increased melanin (melanosis)
(ii) hypopigmentation: skin colour that is paler than normal but not completely white (often associated with loss of melanin).
(iii) erythema: skin redness due to increased blood supply. It might occur with any skin injury, infection, or inflammation.

For a detailed survey, the interested reader is referred to [10].

## 3. Colour Moments

Colour moments characterize colour distribution in an image. To this aim, first and second moments (mean and variance) are the most commonly used features, although third and fourth moments (skewness and kurtosis) are sometimes employed as well. Higher-order moments are usually not part of the colour moment feature set, because, to obtain a good estimate of their value, more data is required (also, they are more sensitive to noise). Instead, some authors include other statistical measures such as maximum, minimum, range, and entropy alongside colour moments. These simple statistical parameters are easy to compute and have been used extensively in dermoscopy image analysis literature [11–35].

Colour moments are usually calculated for each colour channel separately over a region or the entire image. They can be computed for any colour model. Some authors combined different colour spaces to enrich the colour moment feature set. Ganster et al. [30], for instance, employed the minimum, maximum, average, and the variance of the RGB and HSI colour channels as colour features.

Colour moments have been extensively used for image retrieval [36–39]. The main advantage of using colour moments is that there is no need to store the complete colour distribution [40]. This speeds up image retrieval since there are fewer features to compare. Also, colour moments are scaling and rotation invariant. On the downside, these features are most useful when they are calculated to represent a homogeneous region. They are not often enough to represent all the colour information of an image.

## 4. Colour Histogram

A colour histogram represents the distribution of the composition of colours in an image. It quantizes a colour space into different “bins” and counts the frequency of pixels belonging to each colour bin. In general, a histogram is a representation of the distribution of “continuous numerical data,” like image intensities, where data is grouped into ranges (i.e., bins). When used as an image feature, a histogram is a vector with as many elements as the number of bins, and each element is the count of pixels that “fall” into the corresponding bin.

Colour histograms have been widely employed in dermoscopy image analysis literature. For instance, Xie et al. [26] used a multivariate histogram to capture the colour variegation of lesions. In their method, the RGB colour space was quantized into 16 × 16 × 16 bins and the joint distribution of RGB data was represented via a 3D histogram. In addition, Xie et al. captured the colour differences between lesion and healthy skin surrounding it and used that as an image feature. To this aim, they computed the Euclidean distance between the histogram of lesion and the histogram of peripheral area around the lesion. The latter was carried out using 3D histograms with 4 × 8 × 8 bins in CIE Luv colour space. Other notable studies include the works of Stanley et al. [41], Celebi et al. [11], Rahman and Bhattacharya [33], Situ et al. [42], Ruela et al. [12, 43], and Barata et al. [22, 24, 44].

The formation of a histogram involves various principles. For instance, one can choose to have fixed or variable bin width. One can use “hard assignment” where intensity values are assigned to the closest bin or “soft assignment” instead, where each pixel contributes to all bins based on the distance to all bin centers. For a colour histogram, there are other considerations. For example, one can choose to create a histogram for each colour channel and then concatenate these into one feature vector. This format is often called unidimensional or univariate histogram. Alternatively, one can choose to model the joint distribution of colour channels and create a large histogram that accounts for every combination of triple colour values. The latter is called a multidimensional or multivariate histogram. A colour histogram does not contain pixels' spatial information. The advantage is that since a histogram does not provide spatial information, it is rotation and translation invariant. The disadvantage is that two images with the same colour content but different spatial distribution of these colours are indistinguishable based solely on colour histogram comparisons.

## 5. Colour Asymmetry

Asymmetry is a feature inspired by the clinical diagnosis guidelines. In the ABCD rule of dermoscopy [45], asymmetry is given the highest weight among the four features. In the Menzies method [46], symmetry is one of the two features that rules out malignancy. In the Chaos and Clues algorithm [47], “chaos” is defined as “asymmetry of structure or colour.” Asymmetry is also considered in the CASH algorithm [48] and the three-point checklist [49] guideline. During the Consensus Net Meeting on Dermoscopy [50], asymmetry was identified as one of the three criteria (the other two were atypical pigment network and blue-white structures) that are especially important in distinguishing malignant from benign pigmented skin lesions, with a good interobserver agreement between experts.

In the ABCD rule of dermoscopy, the lesion is bilaterally segmented by two orthogonal axes that are positioned to produce the lowest possible asymmetry score. If both axes show asymmetric contours, internal colours, and dermoscopic structures, the asymmetry score is two. If there is asymmetry on one axis only, the score is one. If asymmetry is absent with regard to both axes, the score is zero. Stolz et al. [45] highlighted that 96% of melanoma cases in their study had an asymmetry score of 2 whereas only 24% of benign lesions showed two-axis asymmetry.

Most authors focused on lesion shape (contour) to detect, measure, and quantify asymmetry. Others tried to include colour and texture in this process as well. Incorporating colour and structures are very crucial to the evaluation of lesion asymmetry since some early melanomas have symmetrical shapes [51]. In the following, we will first, briefly, review the methods that attempted to evaluate the asymmetry of shape. It will be followed by a more detailed review of methods concerned with asymmetry of colour in dermoscopy images.

### 5.1. Asymmetry of Shape

There have been many approaches proposed to quantify the asymmetry of lesions through analysis of lesion contour. Most of these methods try to imitate the ABCD rule of dermoscopy where asymmetry is quantified with respect to a symmetry axis that bisects the lesion. In automatic image analysis approaches, the symmetry axis is determined in a variety of ways, such as principal axes (moment) of inertia of the lesion shape [52]. The asymmetry is then quantified by overlapping the two halves of the lesion along the symmetry axes and dividing the nonoverlapping area differences of the two halves by the total area of the lesion [52–56].

### 5.2. ABCD Mimickers

Pellacani et al. [57] tried to reproduce the asymmetry evaluation approach of ABCD rule of dermoscopy [45] with focus on asymmetry of pigment distribution. They used principal inertia axes of lesion shape as the symmetry axes and quantified pigment distribution asymmetry based on the comparison of the difference in lightness (average grey-level values) between the two halves of the lesion along the two axes. A score of one was attributed to each axis if the grey-level differences were greater than a (empirically defined) threshold. Pellacani et al. evaluated the performance of their proposed approach by comparing it to the performance of two experienced clinicians (interobserver agreement between experts, as well as how their evaluation was combined for comparison to computer, is not described in the paper) over a set of 331 dermoscopic images (113 melanomas and 218 melanocytic nevi). They concluded “human and computer concordance was good for shape asymmetry.” The human/computer concordance for pigment distribution asymmetry was lower than that observed for shape symmetry. Also, pigment distribution asymmetry was found as “the most striking parameter for diagnosis of melanoma (for both clinician and the computer).”

Seidenari et al. [58, 59] modified the method by Pellacani et al. In their work, a grid is laid on the image plane and aligned with the principal inertia axis. Next, colour distance between each block and its corresponding one with respect to each symmetry axis was computed. Each image block was represented by mean and standard deviation of the distribution of CIE LAB colours within the block, and the colour difference was computed using the Bhattacharyya distance [60]. To calculate a measure of symmetry, the colour differences between corresponding blocks were averaged for each axis. A score was automatically attributed to each axis: 0 if average colour distance was equal to or greater than a (empirically defined) threshold and 1 if lower. Comparison with human performance was carried out (similar to that reported in Pellacani et al.) over a set of 459 dermoscopic images. Authors reported that “81% of symmetric/asymmetric evaluations were concordant between clinician and computer.”

Ruela et al. [12] extended the approach of Seidenari et al. and studied the role of colour symmetry in automatic detection of melanoma. In addition to using the mean colour to represent each block (as originally conducted by Seidenari et al.), they used two additional descriptors: (i) unidimensional colour histograms and (ii) generalized colour moments [61]. Ruela et al. also experimented with multiple colour spaces. Moreover, instead of averaging colour difference (as in Seidenari et al.), the colour symmetry of the whole lesion is characterized by statistical properties of the colour distances (maximum, minimum, mean, and variance) which represent the distances between all symmetrical blocks.

Ruela et al. employed these features for classification of melanoma versus benign lesions on a set of 177 dermoscopy images. They experimented with a combination of different classifiers and features. The best results were achieved with the HSV colour space and the kNN classifier. Interestingly, the best colour descriptor was the mean colour vector (the original approach of Seidenari et al.) although the unidimensional colour histogram (computed by concatenating histograms of all colour channels) also achieved good results. This study is particularly insightful concerning their methodology and evaluation efforts. It is, however, open to criticism on three grounds: (i) lack of comparison to prior work, (ii) using a small data set, and (iii) negligence in experimenting with proposed features for discriminating between symmetric versus asymmetric lesions.

Celebi et al. [11]'s paper is yet another study that imitates the ABCD rule of dermoscopy, although they devised a new measure to quantify shape and colour asymmetry. In their approach, the image is rotated (clockwise) to align its coordinate (*x* and *y*) axes with principal inertia axis of the lesion. The lesion is then hypothetically folded around the *x*-axis and the area difference (*A*_*x*_) between the overlapping folds was taken as the amount of asymmetry on the *x*-axis. The same procedure was performed for the *y*-axis. Two asymmetry measures were calculated from *A*_*x*_ and *A*_*y*_ as follows:

(1) A1=min⁡Ax,AyA×100%A2=Ax+AyA×100%.

For colour asymmetry, the same procedure is carried out except that, instead of area, pixel values were incorporated in the calculations: the sum of the absolute grey-level difference between the corresponding pixels in the two folds. The two asymmetry measures noted above (*A*_1_ and *A*_2_) are computed for each image channel, in the RGB colour space, resulting in the total of six-coefficient (6D) colour asymmetry feature. Celebi et al. used shape and colour asymmetry features (along with other features) for classification of dermoscopy images. Experiments using support vector machine (SVM) classification, on a set of 564 images, yielded specificity and sensitivity of about 93%. The paper neither evaluates the proposed asymmetry measure for detection and quantification of lesion shape/colour asymmetry nor reports the efficacy of using this feature (alone) for the main task of lesion classification (diagnosis).

Zortea et al. [62] developed an image analysis system for detection of malignant melanoma from dermoscopy images. The focus of their work was on engineering novel features, including features to quantify asymmetry of lesion shape and colouration. To that aim, they created three features that are described as follows:

(i) *Asymmetry of Shape.* A coordinate system is placed on lesion's center of gravity. For each axis, the difference of lesion area bisected by the axes (i.e., Δ*S*_*i*_ and Δ*S*_*j*_) is computed (e.g., Δ*S*_*i*_ = ‖area(*A*_1_ ∪ *A*_2_) − area(*A*_3_ ∪ *A*_4_)‖) and normalized by dividing by the total lesion area. The coordinate system is then rotated in steps of 10°. The axis with the lowest average Δ*S* is kept as the axis of symmetry and associated *f*_1_ = Δ*S*_*i*_ and *f*_2_ = Δ*S*_*j*_ as asymmetry of shape features.
(ii) *Asymmetry of Colour Intensity.* The computation of this feature is similar to the calculation of shape asymmetry except that intensity values are used, instead of area, to compute *f*_3_ = Δ*C*_*i*_ and *f*_4_ = Δ*C*_*j*_. For example, Δ*C*_*i*_ = Σ_*x*=0_^255^‖*F*_*A*_1_∪*A*_2__(*x*) − *F*_*A*_3_∪*A*_4__(*x*)‖, where *F*(*x*) is the estimated distribution of the 256 grey-level (*x*) values from the pixels belonging to either side of an axis, and it is computed using Gaussian kernel density estimate.
(iii) *Asymmetry of Colour Shape.* Zortea et al. used grey-level thresholding to segment the lesion. The center of the lesion is defined by computing the center of mass of the binary mask of the lesion. By applying different thresholds to the grey-scale values inside the lesion at percentiles *t* = [0.10,0.20,…, 0.90], different binary masks can be generated. Each would result in a different center of mass. Zortea et al. created a vector **v** whose elements were the Euclidean distances between the detected lesion centers for different threshold values. These distances were normalized by dividing by a lesion-dependent constant defined as the radius of a circle of the equivalent area as the original binary mask. Finally, average and standard deviation of **v** were used as features (i.e., *f*_5_ = *μ*(**V**) and *f*_6_ = *σ*(**V**)).

### 5.3. Classification instead of Feature Engineering

Vasconcelos et al. [63] trained different classifiers (Bayes, SVM, kNN, boosting, and Random Tree) to discriminate between symmetric and asymmetric lesions on two sets of 382 dermoscopy images (227 symmetric and 155 asymmetric) and 80 images (17 symmetric and 63 asymmetric) captured using a handheld mobile smartphone. To that aim, a total of 310 low-level features (including shape, colour, and texture) were extracted from each image. Some of these features were extracted from the entire lesion while other features were extracted from the intensity differences around the axis of inertia (like the method of Celebi et al. [11]). For example, mean and standard deviation of intensity differences were computed for each axis and each channel of RGB, HSV, and CIE LAB colour spaces, as part of colour features.

The study by Vasconcelos et al. is interesting since, unlike others who focused on feature engineering, it has mainly used low-level features and focused on classification for symmetric/asymmetric discrimination. Vasconcelos et al. also applied different feature selection techniques. The best classifier for the dermoscopic image set was the Random Forest classifier, which achieved 83% sensitivity and 89% specificity using as few as eight features. The paper, however, did not indicate which features; a reader is particularly left with the question whether the features extracted from the entire lesion or those extracted from the intensity differences around the axis of inertia were useful. These are only a few of many issues of which this study suffers. Among the other issues, the most serious is perhaps lack of comparison to prior art.

### 5.4. Asymmetry as Noise

Schmid-Saugeon et al. [53, 64] proposed a measure to quantify symmetry based on the mean squared error (MSE) between the original image and the reflected image for any potential axis of symmetry. These authors argued that any object could be decomposed to symmetric and asymmetric components. Considering the asymmetric component as symmetry noise, asymmetry can be measured using peak noise-to-signal ratio (PSNR = 10 log_10_⁡(max_*I*_^2^/MSE), where max_*I*_ is the maximum possible pixel value of image *I*, e.g., max_*I*_ = 255 for an image represented by 8 bits per pixel). A symmetry measure can be defined [64] as

(2) $$\begin{array}{c} \psi \left(\;\varphi ,r,I\;\right)=1-\left(\;\frac{1}{1+PSNR}\;\right), \end{array}$$

where angle *φ* and offset *r* are parameters of any line (potential symmetry axis) in polar coordinates with respect to the center of lesion. The symmetry measure *ψ* is equal to 1 only if symmetry is perfect and decreases to zero for increasing asymmetry. A lesion's axis of symmetry is any line (*φ*_*l*_, *r*_*l*_) that produces *ψ*(*φ*_*l*_, *r*_*l*_, *I*) = 1. The asymmetry score of the lesion is the one that maximizes the symmetry measure (i.e., *ψ* = max_*φ*,*r*_⁡*ψ*(*φ*, *r*, *I*)) or in other words minimizes the mean squared error. The score and optimal parameters can be found through optimization, or by grid searching the parameter space at predefined intervals. The latter approach is practiced by the authors and visualized in what they called “the lesion symmetry map.”

The MSE is computed only for pixels within the lesion. Each pixel is represented using a feature vector consisting of *u* and *v* (of CIE Luv colour space) components. Schmid-Saugeon et al. also used Gabor texture features. Schmid-Saugeon et al. did not conduct an objective experiment to evaluate the effectiveness of proposed feature to quantify asymmetry. However, the proposed asymmetry score was used as a feature to classify melanoma versus benign lesions, on a set of 100 dermoscopy images with results showing 78% sensitivity and 90% specificity. These results were achieved using a linear classifier trained in a 6D feature space consisting of asymmetry score using texture, colour, and shape (for shape asymmetry, instead of intensity values, MSE of the area was computed) (for each of these, asymmetry score corresponding to two axes was computed to mimic ABCD rule). Interestingly, texture asymmetry is reported to be the most discriminant feature although the combination of colour-shape-texture produced the best results (the paper did not indicate the discriminative power of colour (asymmetry) feature).

### 5.5. Normalized Colour Distance

Clawson et al. [65] proposed a method to quantify colour asymmetry and also to visualize (display) it graphically. Their proposed algorithm is based on analysis of pigment distribution along radial paths from the lesion centroid to sample boundary points. For each radial path, the average greyscale value *Av*_*i*_ is computed (where *i* = 1 ⋯ *N* indexes sample points on lesion boundary). A measure is defined as Normalized Colour Distance (the naming convention is rather inappropriate since the measure does not represent the difference or distance between two colours, in a metric of interest, as it is commonly defined in colour science) NDC_*i*_ = *Av*_*i*_/*A*_*L*_ × 100, where *A*_*L*_ is the mean *Av*_*i*_ for the whole lesion (i.e., *A*_*L*_ = ∑*Av*_*i*_/*N*). The NCD values were used to generate a new contour, the shape of which is indicative of pigment distribution within the lesion. For a homogeneous lesion with perfect colour symmetry, *A*_*L*_ = *Av*_*i*_; ∀*i*; thus NCD_*i*_ = 100; ∀*i*, and new contour will be a circle. Hyperpigmented areas (dark pigmentation relative to lesion average colour) will result in larger (NCD_*i*_ > 100) values, and hypopigmented areas will result in smaller (NCD_*i*_ < 100) values.

Clawson et al. derived a number of features from their proposed NCD measure and resultant symmetry contour representation. For example, since a perfectly colour symmetric lesion has a circular contour, the circularity index of the contour might be used to quantify the degree of asymmetry. The scatter plot of NCD values can also be studied for asymmetry analysis since NCD values for a perfect colour symmetric lesion would lie on a line. Deviation from the symmetry line can be used to quantify colour asymmetry.

Clawson et al. [65]'s study is one of the very few studies that has reviewed the literature; although it did not objectively compare their proposed method to the state-of-the-art one, it has cited other studies pertaining to the subject. This study is also the first to engineer a novel feature and to objectively evaluate it for detection of colour asymmetry. However, the method is tested on a set of 30 dermoscopy images with as few as eight positive samples. Therefore, we must be conservative when evaluating the effectiveness of the proposed feature. Also, interestingly, among all the proposed features, the decision tree (with the C5 algorithm) picked only one feature, named LTA/LTW in the paper, as the only significant parameter to differentiate between symmetrical and asymmetrical lesions (i.e., classification can be done by thresholding over this feature alone). The LTA/LTW feature is defined using the curve (scatter plot) of NCD values. It is computed by dividing the largest* trough* (the lowest point the curve sinks to) to the total area under the curve. It represents, in a way, the intensity and area of hypopigmented areas within the lesion. Hypopigmented areas represent melanoma-specific features such as regression. Therefore, one can question whether it is the colour asymmetry or presence of this feature that is being captured by the proposed method.

### 5.6. Reflectional Asymmetry in Histograms

Liu et al. [66, 67] proposed a method to quantify the shape and pigmentation asymmetry of skin lesions. The proposed method comprises many components, and a full review would be overlong. The description of the method's integral component, Reflectional Asymmetry in Histograms, follows. The centroid of the lesion is detected by averaging its spatial information. The lesion area is equally divided into 360 segments around the polar coordinate of its center. Each segment is represented by the area of that segment relative to the total area of the lesion. The relative area values (from 360 segments) can be plotted (e.g., from 0 ⋯ 2*π*) to generate a 1D histogram (where each bin value corresponds to one segment value). Assuming one segment as the bilateral symmetry axis each time, the histogram is populated such that the selected segment is always at the center (in bin #180). The asymmetry of a lesion can then be quantified by minimizing the sum of the Euclidean distances between corresponding bins from the left and the right halves of the histograms. In order to measure pigmentation (colour) asymmetry, instead of the relative area, one can choose, for example, (relative) average intensity values from any of *R*, *G*, or *B* image channels. Instead of these image channels, Liu et al. decided to use melanin index (MI) and erythema index (EI) to “enhance the characterization of pigmentation distributions.” The MI and EI are approximated from *R* and *G* channels according to Takiwaki et al. [68]. Moreover, instead of intensity values, Liu et al. employed a metric called global point signature (GPS) that represents “a point as a vector of scaled eigenfunctions of the Laplace-Beltrami operator computed on the object's surface [69].” Liu et al. computed the GPS representation over their proposed Lesion Pigmentation Elevation Models. The elevation models were created by mapping MI and EI values as the height information (on the *z*-axis) on the image plane. Liu et al. claimed, although not conclusive from the paper, that GPS representation “simultaneously integrates the shape and pigmentation information.” The performance of the proposed asymmetry analysis was tested on 351 dermoscopy images (88 melanomas and 263 benign) for classification of melanoma versus benign lesions. The efficacy of the proposed method for discriminating between symmetric and asymmetric lesions remains to be tested. Interestingly, according to the authors' experiments and despite their laudable effort, the use of GPS representation, as well as employing MI and EI images, only offered a marginal improvement.

### 5.7. Summary and Discussion

Colour asymmetry is one of the most studied topics, compared to other colour features. Although many different techniques are proposed, most of the approaches are mimickers of the clinical ABCD rule of dermoscopy. The most successful method is probably the method of Zortea et al. [62]. Among the other techniques, the most notable are the methods of Schmid-Saugeon et al. [53, 64] and Clawson et al. [65] for their novelty. A list of the papers reviewed in this section is given in Table 1.

## 6. Colour Variegation

In the jargon of dermatology, variegated colouring is the term often used to describe a lesion with multiple colours. Benign lesions tend to have few colours, whereas melanomas often have many. The importance of this observation is reflected by the fact that most diagnosis guidelines [45–48] consider this feature as a sign of malignancy. In contrast, the presence of only a single colour is often enough to exclude the diagnosis of melanoma [46, 47]. Note that the dermatoscope reveals a wider range of colours than those that can be seen with the naked eye.

In clinical practice, the most common way to quantify colour variegation is to count the presence of certain colours in the lesion. In the ABCD rule of dermoscopy [45], for instance, the following six colours are considered important: light brown, dark brown, black, red, white, and blue-grey. Each of these colours in a lesion is assigned one point. The CASH algorithm [48] and Menzies's method [86] follow the same scoring principles (in the Menzies method, the colour “white” is not scored while blue and grey are considered two colours (instead of a blue-grey colour)). Due to this diagnostic importance, the analysis of lesion colouration has been undertaken in several studies. One of the simplest approaches is to derive basic descriptive statistics from the distribution of colour primaries inside the lesion. Seidenari et al. [70, 71] divided each image into nonoverlapping blocks where each block was represented by the average colour of its contained pixels. This was followed by computing colour difference between blocks using the Euclidean distance in the RGB colour space (each colour block is compared with every other block in the image and not only to its corresponding one about the axis of symmetry). Mean, variance, and maximum colour differences were used as measures of colour distribution capturing colour variegation within the lesion. The intuitive idea behind Seidenari et al.'s findings is that a significant difference between RGB values means that the lesion's structure (colours/patterns) is nonhomogeneous. However, there are two drawbacks with this method. First, it is sensitive to the choice of block size. Second, larger colour differences do not necessarily mean more colour variegation. Seidenari et al. performed discriminant analysis over a set of 229 lesions and concluded that their proposed numerical parameters to capture colour variegation (i.e., mean, variance, and maximum colour block distances) are “reasonably” discriminative (higher in melanoma compared to nevi).

Abbas et al. [54] used the *k*-means clustering [87, 88] algorithm to quantize the dominant colours within a lesion. The corresponding percentage of occurrence of each colour cluster is used as features to capture colour variegation (Abbas et al. [54] are not very clear on how the feature in discussion was formed or on how it was used). A major issue with clustering is how to decide the number of clusters. Abbas et al. chose *k* = 6 to imitate the ABCD rule [45]. This means the algorithm considers every lesion to be made up of six colours, even a lesion that is homogeneous with uniform colouration.

Andreassi et al. [89] described an image processing and pattern analysis software (http://www.ddax3.com/eng/index.html) which extracts “colour islands” (colour clusters within the lesion) among other image features. The details of this process were not disclosed (perhaps due to patent protection). Siedenari et al. [90] noted that “colour islands [89] inside the lesion were described by polar moments of inertia (on the *x* and the *y* axes) of different order.” From colour islands, several features were extracted [89] to represent the colour distribution of a lesion. Among these were, for example, the percentage of the island area (with respect to the lesion area) and distance from the center of the colour island to the center of the lesion. These features were studied with different agenda as these authors continued to investigate various skin diseases [55, 56, 91–104].

Celebi et al. [11] devised the feature “centroidal distances”: the distance between the geometric centroid of a lesion and the brightness centroid. The former was computed from the binary lesion mask whereas the latter, the center of gravity of pixels in the lesion, was computed for each colour channel of the RGB space (and for five other colour spaces). To achieve invariance to scaling, the distance values were divided by the lesion diameter. The centroidal distance (for each colour channel) will be small if the lesion colouration is homogeneous because the brightness centroid will be close to the geometric centroid. Note that a symmetric lesion comprised of many concentric colour zones would (potentially) have the centroidal distance of zero just as a symmetric lesion made of a single shade.

### 6.1. Summary and Discussion

The methods described in this section can be seen as different instances of two broader approaches. Either “colour clustering” is used or “colour distribution” inside the lesion is considered to capture colour variegation (Table 2). Among these, only Umbaugh et al. specifically conducted experiments about identification of colour variegation. Other authors focused on discriminating melanoma versus benign lesions. Among those, only Seidenari et al. examined the discriminant power of their proposed feature for differentiating between melanomas and benign lesions (common and atypical nevi).

Colour variegation, although easy to define, can be difficult to identify and subjective to quantify since human capability to distinguish colours is varied and limited. Ideally, image analysis algorithms would identify this effect and measure it objectively and quantitatively to support clinical diagnosis. A major limitation of current state-of-the-art methods (Table 2) remains in that they are blind to “what” colours the lesion is comprised of. To address this defect, some researchers focused on colour classification. The next section is devoted to those methods.

## 7. Colour Classification

The objective of colour classification is to assign labels (such as colour names) to each region (or pixel) of the image using the colour information contained in that region. There are two class of approaches to achieve any classification task: generative models versus discriminative models. Generally speaking, a generative algorithm models data directly from the training samples and performs classification based on which class is most likely to generate the sample according to the model. A discriminative algorithm, on the other hand, models the differences between classes, for example, by finding decision boundaries, and simply categorizes a given sample according to those boundaries (this is an overly simplified description; for a more rigorous definition, refer to [105, 106]).

### 7.1. Discriminative Models

One of the easiest methods for colour classification is to define the boundaries of each colour class in a specific colour space. Sboner et al. [13, 14, 73] classified colours within lesions to four categories of dark brown, light brown, reddish, and whitish veil, in HSV colour space, based on this simple principle. For example, a pixel with Hue ∈[13,157] and Saturation ∈[120,255] and Value ∈[0,137] was classified as dark brown. The boundary values were defined empirically. The success of this method is subject to the separability of colour classes in a given colour space.

Silva et al. [77] performed separability analysis of colour classes on dermoscopy images. They analysed the distribution of six different colour classes (white, black, red, blue-grey, dark, and light brown) in RGB space, using a set of 30 images, and concluded “a suitable classifier would potentially produce good results, with the exception of classes black and blue-grey that cannot be properly distinguished.” Their findings, however, are limited by the fact that their training sample was confined to one annotated image (Silva et al. also considered using six annotated images for training although reportedly “this increased the dispersion of colour classes in the RGB space”).

Seidenari et al. [71, 74, 75] quantitatively evaluated their colour classifier. In their study, two clinicians independently annotated colour regions with colour names on a set of 30 dermoscopy images. From these annotated images, the average RGB values of each region were stored as an example (shade) of that colour name (class), permitting a total of 98 samples compromising a “colour palette” of six colour classes. Seidenari et al. evaluated their work on a database of 331 images (113 melanoma and 218 nevi). For each image, the two clinicians recorded the presence of each colour class. This assessment was compared with the computer's performance. The correlation between clinicians' evaluation and computer's evaluation was reported between 0.73 (for light brown) and 0.893 (for black).

Seidenari et al. also counted the number of colours in each image with results comparable to the evaluation of clinicians. They have also analysed [76] the distribution of colours in melanoma versus (atypical and common) nevi. Results showed that the number of colours in melanoma is higher than that in nevi; in particular, black, blue-grey, and white colours were more frequently found in melanomas. They concluded colour assessment can contribute to the distinction of melanoma, in particular from atypical nevi that share some dermoscopic features with the deadly disease. It is to be noted that labelling each pixel of each image in the nearest neighbor fashion (as practiced by Seidenari et al. and Alcon et al.) could be computationally expensive. Also, using Euclidean distance metric in RGB space for computing colour differences is a refuted practice for colour matching because RGB is not a perceptually uniform colour space. That is, colour differences in RGB do not agree consistently with human visual perception.

### 7.2. Generative Models

Chen et al. [72] labelled each pixel as a melanoma colour or a benign colour. Their labelling algorithm was based on histogram models. Two histograms were populated for each class (melanoma and benign) where each bin of each histogram stores the count associated with the occurrence of the bin colour in the training data for that class. The histogram counts are normalized to sum to unity, converting the histogram to (discrete) probability density function. Thus, for each class, a bin value corresponds to the likelihood of the bin colour belonging to this category. Pixels are labelled by comparing the likelihood values of their associated histogram bins. The downfall of histogram models is that classification accuracy is subject to the representativeness of training data. A pixel is labelled, for instance, as “melanoma colour” if that particular colour was observed more in melanoma training data than that of benign. Chen et al. labelled pixels as “unpopulated” if the colour was not seen in the training data (empty bin) or as “uncertain” if the likelihoods of belonging to each class were approximately equal.

Barata et al. [78] trained a Gaussian Mixture Model (GMM) for classification of five different colour classes (black, blue-grey, white, dark, and light brown) and compared its performance with that of a dermatologist. Unlike histogram models, GMMs can be made to generalize well on small amounts of training data at the cost of tuning their parameters. In their study [78], colour regions were manually segmented and labelled by a dermatologist for a training set of 27 dermoscopy images. For each colour class, small patches were randomly extracted from the corresponding segmented colour regions. The average value of HSV and LAB colour components (a 6D feature vector) from each patch is taken to represent a training sample. Barata et al. trained a GMM for each colour class using this training data. The overall performance was evaluated on a test set of 103 images (27 melanoma and 76 nevi). The objective was to determine presence/absence of each colour class in images. Test images were decomposed into small patches. Each patch was represented by average (HSV and LAB) colour components. Using this feature and the GMM models, the patch was labelled by comparing the likelihoods that GMMs generated. Reported results indicate average Spearman correlation of 0.7981 with the performance of human expert [78].

### 7.3. Summary and Discussion

All of the studies reported in this section employed colour classification to quantify the number of colours in images (lesions). As previously discussed, this is to imitate clinical practices (such as ABCD rule) to quantify colour variegation feature of skin lesions. Among these studies, many did not directly evaluate the performance of their proposed colour classification method. Instead, they performed classification of melanoma versus benign lesions. Most studies used nearest neighbor technique in RGB colour space and define six colour classes (see Table 3). Lack of benchmarking for comparison and evaluation is a major challenge that remains to be addressed. The state of the art might be improved by incorporating and adapting existing methods in the context of image understanding for colour naming [107–110]. Colour naming is the action of assigning a linguistic colour label to image pixels and has many applications in the domain of computer vision such as for image retrieval [111], image segmentation [112], image editing [113], image description [114], pattern colourisation [115], data visualization [116], and colour constancy [117].

Among the common colours under dermoscopy, there are a few that are more specific to melanoma. For example, blue-grey or blue-whitish colour is considered a significant indicator of the disease (see 7-point checklist [118], Menzies method [86], and three-point checklist [49]). Therefore, some studies aimed at detecting certain colour and use this characteristic to classify the lesion as melanoma or benign. The next section will cover this topic.

## 8. Colour Detection

The goal is to detect specific colours that have diagnostic significance. For example, detection of blue-white veil colour or hyperpigmented areas. Colour detection is in a sense a binary colour classification. The reasons why a section is dedicated to colour detection, separated from colour classification, are twofold. Firstly, these methods do not aim to assign a class label to every pixel (or every region) in the image. They aim to identify the presence or absence of certain colours (and in some cases to localise those colours). Secondly, these methods might use features other than colour information such as texture and shape. This might seem unnecessary or even inappropriate since the task is indeed colour detection. The reader is reminded however that “colour” here is a specific dermoscopic feature and not necessarily a particular hue. For example, what dermatologist annotate as the blue-white veil is a mixture of many different hues including blue, white, grey, and purple. The methods in this section are grouped together based on the principal approach underlying their colour detection algorithm.

### 8.1. Thresholding

Thresholding is one of the simplest methods of image segmentation. Ogorzałek et al. [15] used thresholding in RGB space to detect white, black, and blue-grey areas. For instance, white areas were identified as pixels which satisfied *R* > 110, *G* > *R* − 26, and *B* > *R* − 20. There is no indication on how these decision rules were generated. Also, the paper does not provide any experiment to evaluate the success of colour detection. The detected colours were quantified by their area as part of a feature set for classification of skin lesions (computer-aided diagnosis).

Pellacani et al. [71, 79] extracted dark areas in dermoscopy images. Their definition of “dark” appears to be dark brown and black pigment areas with irregular shape or asymmetric distribution that are frequently observed in melanomas. To that aim, they introduced two features: absolute dark area (ADA) and relative dark area (RDA). For ADAs, first, the mean brightness of the surrounding skin was computed as a reference brightness level. Next, the ratio between the brightness of each lesion pixel and the reference (skin's) brightness was computed. If this value was lower than a given threshold (empirically set to 0.13), the pixel was considered “absolutely” dark. For RDAs, the histogram of lesion brightness values were divided into four quartiles and the quantile corresponding to the lowest brightness was considered “relatively” dark. To explore the diagnostic importance of these features, statistical analysis was conducted on a set of 339 dermoscopy images (113 melanomas and 226 melanocytic nevi). For this analysis, simple numerical parameters such as region area and average intensity were computed from the detected dark areas for each lesion. Results suggested a statistically significant difference between the two classes (melanoma versus melanocytic nevi).

Sforza et al. [82] proposed an adaptive segmentation of grey areas in dermoscopy images. It seems that by “grey” the authors meant blue-grey (or blue-white) areas. The paper achieved this by thresholding on the B component of HSB colour space. The threshold values are induced “adaptively” although the paper is unclear on how this adaptive process was carried. The paper also lacks quantitative evaluation; results are shown qualitatively for only five dermoscopy images.

### 8.2. Decision Tree

Celebi et al. [80, 81] automatically segmented blue-white veil areas in dermoscopy images. Their approach involved pixel classification using explicit thresholding, where a trained decision tree induced the threshold values. They used a set of 105 dermoscopy images, consisting of 43 images containing sizeable blue-white veil areas with the remaining 62 free of this feature. For each image, a number of small circular regions that contain either veil or nonveil pixels were manually determined by a dermatologist and used for training. A decision tree classifier with C4.5 [119] induction algorithm was employed to classify each pixel in the training stage into two classes: veil and nonveil. Among the 18 different colour and texture features, only two features appeared in the induced decision rules: The classification was conducted by thresholding on a normalized-blue channel (*B*/{*R* + *G* + *B*}) and relative-red feature (defined as $R-{\bar{R}}_{s}$, where ${\bar{R}}_{s}$ is the mean of red channel values for* healthy* skin areas only). Celebi et al. further developed a second decision tree classifier to use detected blue-white veil areas for discriminating between melanoma and benign lesions. The detected veil areas were characterized using simple numerical parameters such as region area, circularity, and ellipticity measures. Experiments on a set of 545 dermoscopy images yielded 69.35% sensitivity and 89.97% specificity.

De Vita et al. [16, 83] detected image regions containing blue-white veil, irregular pigmentation, or regression features. To this aim, first, the lesion is segmented into homogeneous colour regions. Next, simple statistical parameters such as mean and standard deviation are extracted from HSI colour components for each region. Finally, a Logistic Model Tree (LMT) is trained to detect each colour. LMT is a supervised learning classification model that combines logistic regression and decision tree learning. De Vita et al. detected these colour features as part of their system [16] for automatic diagnosis of melanoma based on the 7-point checklist clinical guideline. They also evaluated the performance of their colour detection method over a set of 287 images (150 images were used for training and 137 for testing). It is not clear whether the test was aimed to identify (presence/absence) or to localise the colour features. Nevertheless, results were shown with average specificity and sensitivity of about 80%.

### 8.3. Other Methods

Wadhawan et al. [84] detected blue-white veil areas in dermoscopy images. Their method relied on a linear SVM to classify image patches to veil or nonveil. Image patches were extracted over the lesion area using a regular grid sampling. For each image patch, a feature vector was computed by concatenating histogram representation of pixel values in various colour channels of different colour spaces. They evaluated their method by performing 10-fold cross-validation on a set of 489 dermoscopy images (163 containing the veil and remaining 326 free of this feature). For training, images were manually segmented and annotated by one of the authors. For testing, only presence/absence of the feature is considered. Results are reported with an average sensitivity of about 95% and an average specificity of about 70%.

Lingala et al. [85] detected blue areas in dermoscopy images and further classified them to three shades of lavender and dark and light blue using fuzzy sets membership functions. Their colour detection method builds on a simple thresholding approach similar to Ogorzałek et al. [15]. A pixel is considered as “blue” if its normalized RGB values were within a certain range determined empirically (the threshold values were not reported). These blue areas were further classified into lavender and light and dark blue by thresholding their intensity value. This thresholding scheme was used to generate training data using 22 dermoscopy images. The training data was then used to determine the parameters of fuzzy set membership functions for three shades of blue. The method was evaluated over a set of 866 images (173 melanoma and 693 benign). There is no indication of how successful the colour detection was. The evaluation was conducted by classifying lesions to melanoma versus benign, by extracting simple statistical features over blue areas. Interestingly, the effect of using fuzzy set membership versus simple thresholding is evaluated and the improvement in classification is reported to be less than 0.5%. Although the idea of Lingala et al. [85] is interesting, their study suffers from a number of flaws. One example is that since parameters of fuzzy sets were identical for dark and light blue, authors performed thresholding (again) over intensity channel to separate these. This could have been foreseen since intensity thresholding was used to generate training data but for fuzzy set representation, colours were represented in normalized RGB space where the intensity information is discarded.

### 8.4. Summary and Discussion

All the studies reported in this section can be described as using discriminative algorithms. Moreover, these studies follow the classical paradigm of supervised learning that requires extensive annotation of training images using instances of each colour feature. This is difficult (or even impossible) to be carried accurately and consistently due to the subjectivity of feature definition and poor interobserver agreements. Since ground-truth annotations were always made by an expert rather via a consensus of experts' opinions, it is hard to make outright claims about the success of these algorithms especially as all of these studies have failed to provide a comparison to other algorithms.

In terms of categorization, these works could be divided into two categories: pixel-based and region-based methods (see Table 4). Region-based methods often segment the lesion into homogeneous colour regions before further analysis. In all these cases, the emphasis is to use colour features while structural information such as texture is either ignored [15, 16, 83–85] or found futile [80, 81].

## 9. Colour Calibration

For colour image processing, the fidelity of colour throughout image acquisition process is vital. Colour can dramatically change as a result of changes in imaging setup, such as varying illumination or altering acquisition device. Thus, an image processing algorithm that relies on colour information is subject to disruption. In many applications, an accurate colour calibration, therefore, appears to be necessary, to provide an image with reproducible colours, independent of the capturing system and the illuminant characteristics. This is often achieved by finding a relationship between the device-dependent output colour values of a camera (usually in RGB) and a standard colour space (such as the CIE *XYZ* or sRGB). The process usually involves imaging a calibrated target, often a colour checker, and then performing a least-squares regression to find a transformation matrix that maps the camera's RGB of each colour checker patch to their corresponding (standard) colour values. The measurements should be done under a known lighting system (typically D65). If the lighting cannot be controlled, then a measurement of the illuminant irradiance at each patch is needed.

Colour calibration is of double concern for dermatology since the acquisition of repeatable and high-quality images in terms of colour fidelity and resolution is essential for the comparison of time-series images during follow-up studies. However, to date, colour calibration in dermatology has been little investigated. One of the early studies that focused on colour calibration for dermatology was conducted by Haeghen et al. [120] in which a complete setup and calibration of an imaging system for use in dermatology was thoroughly described. Haeghen et al.'s calibration process involved estimation of camera-specific parameters (such as camera offset, colour gain, and aperture) and colour transformation matrix for standardisation. The latter was computed by acquiring images of the Macbeth colour checker chart and determining the relationship between the images and the CIE LAB values of the colour patches, acquired with a spectrophotometer. This calibration was reported to require 5–10 minutes' time of manual effort, which would remain valid for weeks of normal operation.

## 10. Contrast Enhancement

Contrast enhancement, here, refers to increasing visual discrepancy of the lesion from the normal skin surrounding it. This is often practiced as a preprocessing step aiming to improve the task of lesion segmentation. Lesion segmentation is regarded as a crucial step in dermoscopy image analysis that could affect all downstream processes to the final diagnosis. The most trivial segmentation technique is the application of grey-level thresholding methods such as Otsu's [121]. These techniques rely on the assumption that a typical dermatological image consists of two classes of pixels: the lesion and the normal skin. Therefore, the histogram of grey-level values would have two modes. Almost every method that has been proposed in the literature attempts to ensure the histogram of pixel values is bimodal and the concavity between the two modes is maximal.

Among the various proposed techniques, one of the most common practices is selecting a colour channel that maximizes the discrepancy of the lesion from the normal skin surrounding it. Ganster et al. [30], for instance, performed thresholding over the blue channel of RGB for lesion segmentation. Madooei et al. [31, 122] found empirically that using the geometric mean of RGB channels ($\mu =\sqrt[{3}]{R\cdot G\cdot B}$) highlights the lesion from its surrounding and can aid segmentation based on grey-level thresholding. Schaefer et al. [123, 124] used the luminance *L* = *R* + *G* + *B* but also applied Automatic Colour Equalization (ACE) [125] to compensate for poor contrast and lack of colour calibration. The ACE is a colour normalization technique which (in a nutshell) combines two classical techniques of max-RGB and Grey-world normalization (refer to [125] for details.). Celebi et al. [126] created a greyscale image as a weighted sum of the input RGB (*I* = *w*_1_ *R* + *w*_2_ *G* + *w*_3_ *B*; ∑_*i*_ *w*_*i*_ = 1). They carried an exhaustive search over a set of possible *w*_*i*_ values that maximized class separability or histogram bimodality.

Hintz-Madsen et al. [127] employed principal component analysis (PCA) of RGB data. After applying PCA, the first principal component best explains the variance in the image data. Assuming most variation occurs at the edges of the lesion, by retaining the first principal component, we shall have a greyscale of the input image that is optimal for border extraction. Madooei et al. [128] also employed PCA for contrast enhancement although the PCA was carried out in the optical density space of image data: log (RGB). PCA is a simple technique, easy to implement and fast to compute. On the downside, it can be speculated that PCA-based contrast enhancement would be affected by the presence of other structures such as hair or vessels in the image.

Gómez et al. [129] proposed independent histogram pursuit (IHP) that, similar to PCA, linearly transforms the original data into an uncorrelated orthogonal space. However, unlike PCA, which attempts to condense the information (variability) of measured data explained by the first component, IHP specifically aims to find projections in which the lesion and the background (healthy skin) are maximally separated. Their method is iterative and greedy (similar to that of Celebi et al. [126]). The computation of greedy techniques is more cumbersome than PCA-based methods. Gómez et al. [129] and Madooei et al. [128] are among the few who reported experimental procedure and results that showed the improvement of segmentation as a result of contrast enhancement, with a comparison to prior art. It remains to be experimentally confirmed, however, whether contrast enhancement or better segmentation offers an advantage for the task of skin lesion image analysis.

## 11. Attenuation of Shading

Shading is, to put simply, variations in image intensity due to the geometry of the scene (e.g., curvature of object surface). The effect of shading can be caused by other factors such as nonuniform illumination or as the result of a variable gain and offset in camera's sensor. In clinical images, shading is typically caused by imaging non-flat skin surfaces. In dermoscopic images, shading is usually induced by intensity falloff near the edges of the image (natural vignetting around the center of the optical axis). As a result, the image might be bright in the center and decreases in brightness as one goes to the edge of the field of view. In some other cases, the image might be darker on one side or one corner and brighter on the other side. Shading may obscure skin surface details and appear as additional features that are not intrinsic to the lesion. It can also disrupt lesion segmentation. In the literature of skin lesion analysis, most efforts are centered over postprocessing of the image to remove the shading effect.

Norton et al. [130] used adaptive histogram equalization to reduce the shading effect on the green channel before applying grey-level thresholding for segmentation of lesion. Tanaka et al. [131] subtracted each image from a background brightness that accounted for shading due to body curvature. This background image was computed by a moving average operation on each row of image data. Møllersen et al. [132] used the illumination of an empty field as a correction filter (white shading correction). In this technique, a “bright-field” image was captured before imaging the lesions by placing a “white surface” that covered camera's entire field of view in the scene. The intensity values of the image were then divided (or subtracted) from the intensity values of the bright-field image to correct the shading defect.

Madooei et al. [128] attempted to attenuate shading by normalizing the value channel of HSV colour space. They employed a photometric model to form a 1D illumination-invariant image (from sRGB input), called the intrinsic image [133]. The intensity normalization was then induced by matching the histogram of the V channel to the histogram of the intrinsic image.

## 12. Conclusion

A colour image is represented as an array of pixels, where each pixel contains numerical components (usually a triple) that defines a colour according to a colour space. Variety of colour spaces are available; they are often made for different applications (such as for display, printing, colour matching, and television broadcasting). Descriptions of different colour spaces can be found in [36].

Once the colour space is specified, colour features can be extracted from images or image regions. Some studies used pixel values directly as colour features [17, 20, 28, 134] whereas most others employed colour moments (§3) and colour histograms (§4). These are primitive or low-level features that, for example, parametrize the distribution of colour value in an image. A growing trend in recent years is in developing high-level (clinically meaningful) visual features such as colour asymmetry (§5), colour variegation (§6), colour classification (§7), and colour detection (§8).

Among other studies, there are those that focused on “colour quantization” [15, 17, 18, 23, 28, 30, 71, 135, 136] and those aimed at “colour segmentation” [132, 137–141]. Colour quantization is aimed at reducing the number of colours [88]. True-colour images typically contain thousands of colours, which makes their display, storage, transmission, and processing problematic. For this reason, colour quantization is commonly used as a preprocessing step for various graphics and image processing tasks. Most quantization methods are essentially based on data clustering algorithms. Note that colour histogram involves colour quantization.

It is worth noting that few studies [71, 75, 142–144] focused on “colour quantitation,” an attempt to quantify the colour such that it can be objectively and quantitatively measured and compared. It is important to remind ourselves that colour cameras are not built for measurement of colour. They are designed to capture an image of a scene with acceptable appearance for human viewing.

When an image is captured, it may not have the optimal quality for subsequent analysis. Image enhancement is the preprocessing step that serves to compensate for the imperfections of image acquisition. Good performance of the methods at this stage not only ensures correct behaviour of the algorithms in the following stages of analysis but also relaxes the constraints on the image acquisition process. In this review paper, we also focus on those image enhancement techniques that affect the use of colour features: colour calibration (§9), contrast enhancement (§10), and shading attenuation (§11).

## Figures and Tables

**Table 1:** Methods for capturing colour asymmetry.

Authors	Year	Method
Schmid-Saugeon et al. [64]	2000	Asymmetry as noise
Schmid-Saugeon et al. [53]	2003	Asymmetry as noise
Pellacani et al. [57]	2006	ABCD mimickers
Seidenari et al. [58]	2006	ABCD mimickers
Seidenari et al. [59]	2006	ABCD mimickers
Celebi et al. [11]	2007	ABCD mimickers
Clawson et al. [65]	2007	Normalized colour distance
Liu et al. [66]	2011	Reflectional asymmetry
Liu et al. [67]	2012	Reflectional asymmetry
Ruela et al. [12]	2013	ABCD mimickers
Vasconcelos et al. [63]	2014	Classification
Zortea et al. [62]	2014	ABCD mimickers

**Table 2:** Methods for capturing colour variegation.

Authors	Year	Colour clustering	Colour distribution
Seidenari et al. [70]	2005	✓	✓
Seidenari et al. [71]	2006	✓	✓
Celebi et al. [11]	2007		✓
Abbas et al. [54]	2013	✓	

**Table 3:** Colour classification studies.

Authors	Year	Method	Colour space	Number of colour classes
Sboner et el. [13]	2001	Thresholding	HSV	4
Chen et al. [72]	2003	Histogram	RGB	2
Sboner et al. [73]	2003	Thresholding	HSV	4
Seidenari et al. [74]	2003	Nearest neighbor	RGB	6
Pellacani et al. [75]	2004	Nearest neighbor	RGB	6
Sboner et al. [14]	2004	Thresholding	HSV	4
Seidenari et al. [76]	2005	Nearest neighbor	RGB	6
Seidenari et al. [58]	2007	Nearest neighbor	RGB	6
Seidenari et al. [71]	2006	Nearest neighbor	RGB	6
Silva et al. [77]	2012	Nearest neighbor	RGB	6
Barata et al. [78]	2014	Gaussian mixture	HSV & Lab	5

**Table 4:** Colour detection studies.

Author	Year	Method	Approach	Target
Pellacani et al. [79]	2004	Thresholding	Pixel-based	Dark areas
Celebi et al. [80]	2006	Decision tree	Pixel-based	Blue-white veil
Seidenari et al. [71]	2006	Thresholding	Pixel-based	Dark areas
Celebi et al. [81]	2008	Decision tree	Pixel-based	Blue-white veil
Ogorzałek et al. [15]	2010	Thresholding	Pixel-based	White, black, and blue-grey
Sforza et al. [82]	2011	Thresholding	Pixel-based	Grey areas
De Vita et al. [83]	2012	LMT	Region-based	Blue-white veil
Wadhawan et al. [84]	2012	SVM	Region-based	Blue-white veil
Fabbrocini et al. [16]	2014	LMT	Region-based	Blue-white veil
Lingala et al. [85]	2014	Fuzzy sets	Pixel-based	Blue areas
